# Gynæcology

**Published:** 1893-04-01

**Authors:** 


### THE PRACTITIONERS' BOOKSHELF.

### GYNÆCOLOGY.

We cannot say that we are much enamoured of cram books for students, but it appears that everyone is not of our opinion, for a second edition of Dr. Gubb's "Aids to Gynæcology" has been called for.* The causes of disease and the symptoms are plainly, correctly, and systematically arranged in the little book, but the treatment, as must be in such a volume, takes the usual subordinate place. The book being only a cram-book, it would be invidious to point out the numerous deficiencies present, but there are one or two inaccuracies which should not have been allowed to creep into a second edition. Dr. Gubb, for instance, seems to think, to judge from his remarks on page 21, that by dyspareunia—which, by the way, he spells with an *i* here and on page 43 and in the index—is meant impossible sexual connection. The term was introduced by Dr. Barnes to express painful or difficult connection. Again, in the index for "ascites" we are referred to "Tumours of the Uterus," page 84, but on turning to that page we fail to find any mention of that symptom. No doubt this work will be much used by a certain class of students, but we should recommend a larger manual to anyone who wishes to make himself a master of the theories and treatment of the diseases of women.

